# Synthesis, Molecular Docking Studies and In Silico ADMET Screening of New Heterocycles Linked Thiazole Conjugates as Potent Anti-Hepatic Cancer Agents

**DOI:** 10.3390/molecules26061705

**Published:** 2021-03-18

**Authors:** Huda R. M. Rashdan, Mohamed El-Naggar, Aboubakr H. Abdelmonsef

**Affiliations:** 1 Chemistry of Natural and Microbial Products Department, Pharmaceutical and Drug Industries Research Division, National Research Centre, Dokki, Cairo 12622, Egypt; 2 Chemistry Department, Faculty of Sciences, University of Sharjah, Sharjah 27272, United Arab Emirates; melnagrr@sharjah.ac.ae; 3 Chemistry Department, Faculty of Science, South Valley University, Qena 83523, Egypt; aboubakr.ahmed@sci.svu.edu.eg

**Keywords:** thiazoles, 1,2,3-triazoles, anti-hepatic cancer agents, Rho6 protein

## Abstract

Thiazoles are important scaffolds in organic chemistry. Biosynthesis of thiazoles is considered to be an excellent target for the design of novel classes of therapeutic agents. In this study, a new series of 2-ethylidenehydrazono-5-arylazothiazoles **5a**–**d** and 2-ethylidenehydrazono-5-arylazo- thiazolones **8a**–**d** were synthesized via the cyclocondensation reaction of the appropriate hydrazonyl halides **4a**–**d** and **7a**–**d** with ethylidene thiosemicarbazide **3**, respectively. Furthermore, the thiosemicarbazide derivative **3** was reacted with different bromoacetyl compounds **10**–**12** to afford the respective thiazole derivatives **13**–**15**. Chemical composition of the novel derivatives was established on bases of their spectral data (FTIR, ^1^H-NMR, ^13^C-NMR and mass spectrometry) and microanalytical data. The newly synthesized derivatives were screened for their in vitro anti-hepatic cancer potency using an MTT assay. Moreover, an in silico technique was used to assess the interaction modes of the compounds with the active site of Rho6 protein. The docking studies of the target Rho6 with the newly synthesized fourteen compounds showed good docking scores with acceptable binding interactions. The presented results revealed that the newly synthesized compounds exhibited promising inhibition activity against hepatic cancer cell lines (HepG2).

## 1. Introduction 

In the scope of our program, we are aiming to synthesize biologically active compounds from available inexpensive starting materials [1,2,3,4,5,6,7,8,9,10,11,12,13,14,15,16]. Functionalized thiazoles have gained much attention owing to their biological importance [17,18] such as anti-Trypanosoma cruzi agent [19], human adenosine A3 receptor antagonists [20], antiviral [21], HIV-protease inhibitory agents [22], antimicrobial [23], cytotoxic and anticancer agents [24,25]. Compounds possess two thiazole rings either connected through a linker as in bis-thiazoles, or directly connected showed promising biological activity such as DNA replication inhibitors in the cancer cells and HIV-protease inhibitors [26,27]. It was also reported that thiazoles have an anti-biofilm effect against Pseudomonas aeruginosa [28].

Cancer is a disease characterized by uncontrolled cell growth with the potential to invade other parts of the body. Hepatic cancer is the most common type of primary liver cancer, which causes death in people with cirrhosis. The Rho family of GTPases is a family of small signaling G proteins. They are important regulators of cell cycle progression, and are responsible for gene expression [29,30,31]. Homo sapiens Rho6 protein works as a sensitive molecular switch existing either in an active GDP-bound form or an active GTP-bound form. Exchange from GDP to GTP is catalyzed by the guanidine exchange factor (GEF), leading to activation in response to various upstream signals. On the other hand, GTPase-activating protein (GAP) increases the intrinsic GTPase activity, resulting in the inactivation of the protein. The overexpression of Rho6 protein has been found to be increased in some human cancers, including hepatocellular carcinoma (HCC) [32]. Therefore, herein we decided to search for novel thiazole derivatives as anticancer agents based on a computer-aided docking approach. 

## 2. Results and Discussion

### 2.1. Chemistry

**Scheme 1:**
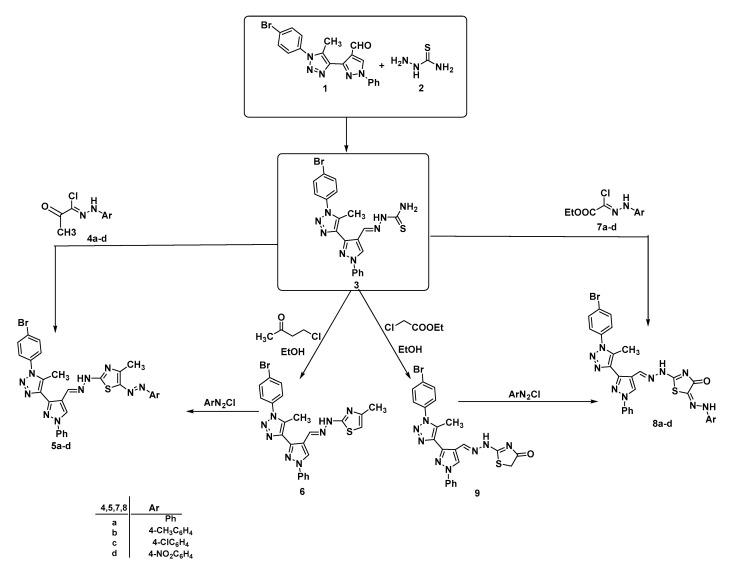
Synthesis of new thiazole derivatives **5a**–**d** and thiazolone derivatives **8a**–**d**.

3-(1-(4-bromophenyl)-5-methyl-1*H*-1,2,3-triazol-4-yl)-1-phenyl-1*H*-pyrazole-4-carbaldehyde **1** was reacted with thiosemicarbazide **2** to give the corresponding ethylidene thiosemicarbazone **3**. We launched our research on the reactions of ethylidenethiosemi carbazone **3** with the appropriate α-keto hydrazonoyl halides **4a**–**d** in dioxane with catalytic amount of triethylamine (TEA) (Scheme 1). The structures of isolated products **5a**–**h** were confirmed by elemental analysis together with spectral data. For example, the IR spectra of the new compounds revealed in each case the absorption bands in the region 3265–3436 and 1590–1610 cm^−1^ owing to the (NH) and (C=N) groups, respectively. In ^1^H-NMR spectra, all the products showed characteristic singlet signals in the region δ 11.34–11.73 ppm (D_2_O exchangeable), referred to as the −NH protons. Based on the demonstrated results, the products isolated from the reactions of **3** with **4a**–**h** can be assigned (Scheme 1).

Authentic samples of **5a**–**d** could be prepared via alternative synthetic pathway. Here, ethylidenethiosemi carbazone **3** was reacted with chloroacetone under thermal conditions to give thiazole derivative **6**. Coupling of the latter product **6** with the appropriate arene diazonium chloride give the respective authentic samples **5a**–**d** (Scheme 1).

In a similar manner, thiosemicarbazone derivative **3** was reacted with ethyl (*N*-arylhydrazono)chloroacetates **7a**–**d** in dioxane in the presence of TEA, affording in each case a single isolable product **8a**–**d**. The structures of **8a**–**d** were elucidated based on spectral data and elemental analysis (see Experimental part). For instance, the IR spectra of the products showed, in each case, one carbonyl band at 1695–1710 cm^−1^ and two NH bands in the regions 3334–3325 and 3259–3250 cm^−1^. Their mass spectra of the latter products revealed in each case, the molecular ion peaks at the expected *m/z* values and their elemental analysis data were consistent with the assigned structures. The thiazolidinone compound **9** was obtained by reaction of the thiosemicarbazone derivative **3** with ethyl chloroacetate in ethanol, in the presence of anhydrous sodium acetate. Coupling of the latter product **9** with arenediazonium chloride in ethanol give products identical in all aspects with the respective authentic samples **8a**–**d** (Scheme 1).

**Scheme 2:**
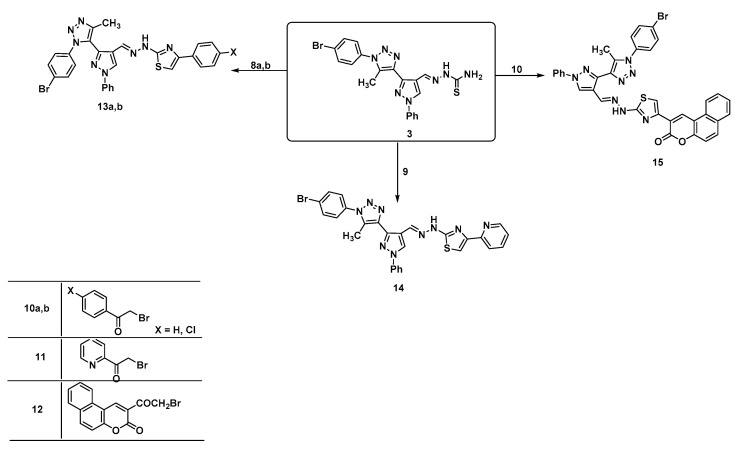
Synthesis of thiazole derivatives **13**–**15**.

On the other hand, the thiosemicarbazone derivative **3** was reacted with different bromoacetyl compounds **10**–**12** to afford the respective thiazole derivatives **13**–**15** (Scheme 2).

### 2.2. Docking Study, SAR Analysis and ADMET Properties

**Figure 1:**
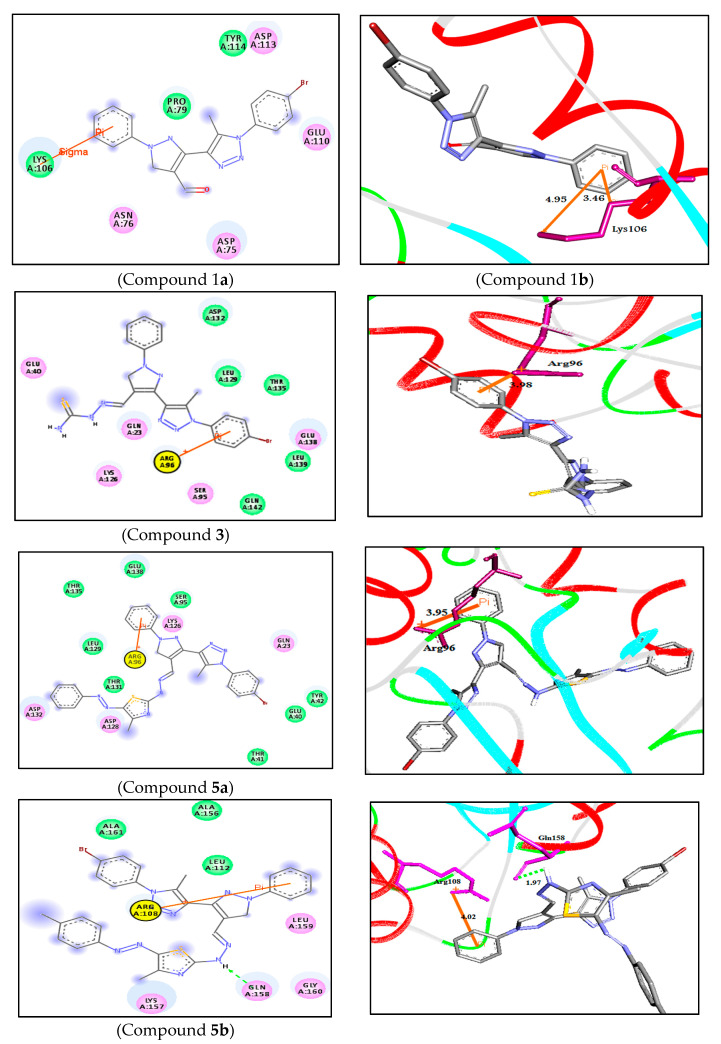
2D and 3D representations of Rho6-compound complexes. Hydrogen bonds are represented in green and blue dotted lines, while π-stacking are shown in orange lines.

The new synthetic compounds were subjected to dock with the active site of Rho6 protein using PyRx-virtual screening software 0.8. The theoretical binding mode of interactions of the fourteen compounds against the binding site was investigated using molecular docking studies. The crystal structure of the human RND1 GTPase in the active GTP bound state (ID: 2CLS) with resolution 2.31 Å was retrieved from RCSB for further study. All water molecules and ligand were removed from the PDB file. The grid box with dimension 25 Å × 25 Å × 25 Å was centered at the active site of the target. Nine conformers for each docked compound were obtained from the docking process, and the conformation with the best scored pose and the lowest binding energy was selected for further study. The docking scores were expressed in negative energy terms, measured in kcal/mol unit, and sorted depending on the higher negative value which implies the best affinity towards the target. The 2D and 3D representations of the non-covalent interactions of protein-compound complex were visualized using Discovery Studio 3.5 [33] as represented in Figure 1. Table 1 contains the docking results, beside the potential interferences (hydrogen bonds, π-π stacking, π-cation and π-sigma), bond lengths between the compounds and Rho6. Compound **1** with binding energy −6.8 kcal.mol^−1^, docked with the target through arene-cation and arene-sigma interactions with Lys106 at the distances of 4.95 and 3.46 Å respectively. In addition, compounds **3** and **14** docked with the residue Arg96 through arene-cation contacts at the distances of 3.98, and 4.02 Å respectively. In addition, compound 14 showed one hydrogen bond interaction with Ser95 at 2.95 For the set of derivatives **5a**–**5d**; the compound **5a** with phenyl ring showed binding energy −8.2 kcal.mol^−1^ docked with the residue Arg96 through arene-cation interaction at the distances of 3.95 Å. On the other hand, introducing of electron donating group as –Me to phenyl ring as in compound **5b,** increases the docking energy to −9.2 kcal.mol^−1^ [34]. Compound **5b** (−9.4 kcal.mol^−1^) exhibited H-bonding and arene-cation interactions with Gln158 and Arg108 at 1.97 and 4.02 Å, respectively. Introducing of electron withdrawing groups on phenyl ring causes lower activity than electron donating groups [34]. For compounds **5c** and **5d** with electron withdrawing groups like −Cl (−9.0 kcal.mol^−1^) and –NO_2_ (−9.1 kcal.mol^−1^), they exhibited H-bonds and arene-cation interactions with the active site of the target. For other set of derivatives **8a**–**8d**; the compound **8a** exhibited two hydrogen bonding interactions with Ser64 and Trp66 at 2.10, and 1.96 Å, respectively. Compound **8b** with electron donating group exhibited high docking score (−9.9 kcal.mol^−1^) showed three H-bonding interactions with Gln158 and Leu159. Compounds **8c** and **8d** with electron withdrawing groups –Cl (weak) and –NO_2_ (strong) exhibited lower scores than compound with electron donating group –CH_3_. For compound **13a**, two H-bonds and one arene-cation interactions were formed with the target through Ser95, Glu138 and Arg96 at the distances of 2.50, 2.15 and 4.10 Å respectively. Meanwhile, the molecular docking of compound **13b** showed one hydrogen bond contact with Asp132 at 2.99 Å. Finally, compound **15** (with the binding energy of −9.2 kcal.mol^−1^) revealed two arene-cation interactions with Lys15 at the distances of 5.74 and 5.50 Å, respectively. The protein-compound interaction maps of 2D and 3D for some molecules are depicted in Figure 1. The other docked molecules with the target are represented in Supplementary Materials as Figure S1.

The pharmacokinetics and physicochemical properties, as tabulated in Table 2, provide a quantitative description of what the human body does to a compound that is administrated. According to Lipinski’s rule of five (RO5), most of the synthesized compounds follow the criteria for orally active drugs. Therefore, they may be considered as potential drug candidates against cancer.

### 2.3. Biological Activity

#### Anti-proliferative Activity

The novel derivatives **5**–**15** were screened for their cytotoxicity against the BALB/3T3 (murine fibroblast) and the human liver carcinoma cell line (HepG2) using doxorubicin as standard drug with IC_50_ value 3.56 ± 0.46 µg/mL in MTT assay. Cytotoxic activities were expressed as the mean IC_50_ of three independent experiments. The results are tabulated in Table 3.

Compounds were tested in concentrations from 100 to 0.1 µg/mL. Nd: no detectable activity in the used concentrations. Concentration of DMSO: 1%.

## 3. Conclusions

In our ongoing efforts to develop novel and potential biologically and pharmaceutically active compounds, this work described an efficient approach for the synthesis of novel thiazole derivatives. They were characterized by IR, ^1^H-NMR, ^13^C-NMR, MS and elemental analysis. An in silico study was carried out to identify the potency of the newly synthesized compounds. The molecular docking study revealed that all the synthesized compounds exhibited good binding energy towards the target Rho6. Overall, the newly synthesized compounds represent encouraging starting points for the development of new drug candidates as anti-hepatic cancer agents.

## 4. Experimental

### 4.1. Chemistry

#### Experimental Instrumentation

All melting points were determined on an electrothermal apparatus and are uncorrected. IR spectra were recorded (KBr discs) on a Shimadzu FT-IR 8201 PC spectrophotometer^1^H-NMR and ^13^C-NMR spectra were recorded in DMSO-d_6_ solutions on BRUKER 400 Chemical shifts are expressed in ppm units using TMS as an internal reference. Mass spectra were recorded on a GC-MS QP1000 EX Shimadzu Elemental analyses were carried out at the Microanalytical Center of Cairo University.

2-((3-(1-(4-bromophenyl)-5-methyl-1*H*-1,2,3-triazol-4-yl)-1-phenyl-1*H*-pyrazol-4-yl)methylene)hydrazine-1-carbothioamide (3) Yellow crystals; (69% yield); mp 168–170 °C (EtOH); IR (KBr): *v*/cm^−1^ 3436 (broad, NH, NH_2_), 3092, 2929 (CH), 1620(C=C), 1595 (C=N); ^1^H-NMR (DMSO-d_6_): δ 2.49 (s, 3H, CH_3_), 7.40(s, 2H, NH_2_), 7.57–8.51 (m, 9H, Ar-H), 8.73(s, 1H, pyrazole-H5), 9.26(s, 1H, CH-aliphatic), 11.51 (s, 1H, NH); ^13^C-NMR (DMSO-d_6_): δ 9.6, 119.9, 123.1, 126.2, 128.1, 129.3, 131.6, 133.5, 134.9, 139.7, 141.3, 143.3, 143.9,170.5; MS *m*/*z* (%): 481 (M^+^, 13). Anal. Calcd for C_20_H_17_BrN_8_S (481.38): C, 49.90; H, 3.56; N, 23.28. Found C, 49.96; H, 3.52; N, 23.25%.

*Reactions* of 2-((3-(1-(4-bromophenyl)-5-methyl-1*H*-1,2,3-triazol-4-yl)-1-phenyl-1*H*- pyrazol-4-yl)methylene)hydrazine-1-carbothioamide (3) with hydrazonoyl halides **4a**–**d** and **7a**–**d**.

*General procedure:* A mixture of 2-((3-(1-(4-bromophenyl)-5-methyl-1*H*-1,2,3-tria zol-4-yl)-1-phenyl-1*H*-pyrazol-4-yl)methylene)hydrazine-1-carbothioamide (3) (10 mmol) and appropriate hydrazonoyl halides **3a**–**h** (1 mmol) in dioxane (15 mL) containing triethylamine (0.1 g, 1 mmol) was refluxed until all the starting materials were consumed (**4a**–**d** and **7a**–**d** as monitored by TLC). Excess of solvent was removed under reduced pressure. The product separated was filtered, dried and recrystallized from the appropriate solvent to give compounds **4a**–**d** and **7a**–**d**. The products, together with their physical constants, are listed below. 

2-(2-(3-(1-(4-bromophenyl)-5-methyl-1*H*-1,2,3-triazol-4-yl)-1-phenyl-1*H*-pyrazol-4-yl) methylene)hydrazinyl)-4-methyl-5-(-phenyldiazenyl)thiazole (5a)

Red crystals; (72% yield); mp 210–212 °C (Ethanol); IR (KBr): *v*/cm^−1^ 3368 (NH), 3072, 2920 (CH), 1620(C=C), 1600 (C=N); ^1^H-NMR (DMSO-d_6_): δ 2.49 (s, 3H, CH_3_), 2.61(s, 3H, CH_3_), 7.39–8.56 (m, 14H, Ar-H), 8.70(s, 1H, pyrazole-H5), 9.29(s, 1H, CH-aliphatic, CH=N), 11.56 (s, 1H, NH); ^13^C-NMR (DMSO-d_6_): δ 9.6, 119.6, 123.5, 126.2, 128.4, 129.3, 131.6, 133.5, 135.9, 139.7, 143.3, 143.6, 147.9, 164.9; MS *m/z* (%): 623 (M^+^, 100). Anal. Calcd for C_29_H_23_BrN_10_S (623.54): C, 55.86; H, 3.72; N, 22.46. Found C, 55.92; H, 3.65; N, 22.41%.

2-(2-(3-(1-(4-bromophenyl)-5-methyl-1*H*-1,2,3-triazol-4-yl)-1-phenyl-1*H*-pyrazol-4-yl)methylene)hydrazinyl)-4-methyl-5-p-tolyldiazenyl)thiazole(5b)

Red crystals; (65% yield); mp 193–195 °C (Ethanol); IR (KBr): *v*/cm−^1^ 3404 (NH), 3080, 2972 (CH), 1615(C=C), 1596 (C=N); ^1^H-NMR (DMSO-d_6_): δ 2.35 (s, 3H, CH_3_), 2.59 (s, 3H, CH_3_), 2.61(s, 3H, CH_3_), 7.44-8.59 (m, 13H, Ar-H), 8.77(s, 1H, pyrazole-H5), 9.30 (s, 1H, CH-aliphatic, CH=N), 11.51 (s, 1H, NH); ^13^C-NMR (DMSO-d_6_): δ 9.6, 9.7, 119.8, 123.7, 126.2, 128.8, 129.2, 131.9, 133.6, 138.6, 144.3, 143.6, 147.8, 167.6; MS *m*/*z* (%): 637 (M^+^, 14). Anal. Calcd for C_30_H_25_BrN_10_S (637.56): C, 56.52; H, 3.95; N, 21.97. Found C, 56.58; H, 3.91; N, 21.92%.

2-(2-(3-(1-(4-bromophenyl)-5-methyl-1*H*-1,2,3-triazol-4-yl)-1-phenyl-1*H*-pyrazol-4-yl)methylene)hydrazinyl)-5-(4-chlorophenyl)diazenyl)-4-methylthiazole (5c)

Red crystals; (65% yield); mp 205-207 °C (Ethanol); IR (KBr): *v*/cm^−1^ 3338 (NH), 3046, 2977 (CH), 1620 (C=C), 1592 (C=N); ^1^H-NMR (DMSO-d_6_): δ 2.58 (s, 3H, CH_3_), 2.60 (s, 3H, CH_3_), 7.40–8.62 (m, 13H, Ar-H), 8.81 (s, 1H, pyrazole-H5), 9.30 (s, 1H, CH-aliphatic, CH=N), 11.62 (s, 1H, NH); ^13^C-NMR (DMSO-d_6_): δ 9.9, 119.8, 123.7, 126.2, 128.8, 129.4, 131.9, 133.8, 138.6, 144.3, 143.8, 147.8, 167.6; MS *m*/*z* (%): 659 (M^+^, 2, 20), 657 (M^+^, 15). Anal. Calcd for C_29_H_22_BrClN_10_S (657.98): C, 52.94; H, 3.37; N, 21.29. Found C, 52.89; H, 3.29; N, 21.25%. 

2-(2-(3-(1-(4-bromophenyl)-5-methyl-1*H*-1,2,3-triazol-4-yl)-1-phenyl-1*H*-pyrazol-4- yl)methylene)hydrazinyl)-4-methyl-5-(4-nitrophenyl)diazenyl)thiazole (5d)

Red crystals; (75% yield); mp 161–163 °C (Ethanol); IR (KBr): *v*/cm^−1^ 3425 (NH), 3051, 2927 (CH), 1620(C=C), 1590 (C=N); ^1^H-NMR (DMSO-d_6_): δ 2.27 (s, 3H, CH_3_), 2.48(s,3H, CH_3_), 7.42–8.57 (m, 13H, Ar-H), 8.76 (s, 1H, pyrazole-H5), 9.31 (s, 1H, CH-aliphatic, CH=N), 11.51 (s, 1H, NH); ^13^C-NMR (DMSO-d_6_): δ 9.7, 119.8, 123.7, 126.2, 128.8, 129.2, 131.9, 133.6, 138.6, 144.3, 143.6, 147.8, 167.6; MS *m*/*z* (%): 668 (M^+^, 6). Anal. Calcd for C_29_H_22_BrN_11_O_2_S (668.53): C, 52.10; H, 3.32; N, 23.05. Found C, 52.15; H, 3.29; N, 23.01%. 

*Alternate method for **5a***–***d****:* 2-(2-((3-(1-(4-bromophenyl)-5-methyl-1*H*-1,2,3-triazol-4-yl)-1-phenyl-1*H*-pyrazol-4-yl)methylene)hydrazinyl)-4-methylthiazole (6). To a solution of thiosemicarbazone **3** (2.40 g, 5 mmol) in EtOH (20 mL), chloroacetone (0.46 g, 5mmol) was added. The mixture was refluxed for 6-8 h (monitored by TLC), and then left to cool. The solid product was filtered off, washed with EtOH and recrystallized from dioxane to afford the thiazole derivative 6 as yellow solid, mp 184–186 °C (AcOH); IR (KBr): *v*/cm^−1^ 3428 (NH), 3056, 2915 (CH), 1620(C=C), 1597 (C=N); ^1^H-NMR (DMSO-d_6_): δ 2.28 (s, 3H, CH_3_), 2.50 (s,3H, CH_3_), 6.37(s, 1H, thiazole-5), 7.40–8.62 (m, 9H, Ar-H), 8.62(s, 1H, pyrazole-H5), 9.30(s, 1H, CH-aliphatic, CH=N), 11.52 (s, 1H, NH); ^13^C-NMR (DMSO-d_6_): δ 9.6, 9.7, 119.5, 122.8, 123.5, 126.2, 128.4, 129.3, 132.6, 133.5, 136.9, 139.7, 143.3, 144.6, 148.9, 162; MS *m*/*z* (%): 519 (M^+^, 27). Anal. Calcd for C_23_H_19_BrN_8_S (519.43): C, 53.18; H, 3.69; N, 21.57. Found C, 52.24; H, 3.65; N, 21.54%.

*Coupling of thiazole 6 with arenediazonium chlorides* To a solution of **6** (0.51 g, 10 mmol) in ethanol (20 mL), cooled to 0–5 °C in an ice bath, was added, portion-wise, to a cold solution of arenediazonium chloride (prepared by diazotizing aniline derivatives (10 mmol) dissolved in hydrochloric acid (6 M, 10 mL) with a solution of sodium nitrite (0.73 g, 10 mmol) in water (5 mL)). After the complete addition of the diazonium salt, the reaction mixture was stirred for a further 30 min in an ice bath. The solid that separated was filtered off, washed with water and finally recrystallized from DMF to give products proven to be identical in all respects (mp, mixed mp and IR spectra) with compounds **5a**–**h** which obtained from reaction of **3** with **4a**–**d**. 2-(2-(3-(1-(4-bromophenyl)-5-methyl-1*H*-1,2,3-triazol-4-yl)-1-phenyl-1*H*-pyrazol-4-yl)methylene)hydrazinyl)-5-(2-phenylhydrazono)thiazol-4(5*H*)-one (8a)

Yellow solid; (61% yield); mp 160–162 °C (Ethanol); IR (KBr): *v*/cm^−1^ 3433, 3253 (2NH), 3051, 2922 (CH), 1685 (C=O), 1619 (C=C), 1595 (C=N); ^1^H-NMR (DMSO-d_6_): δ 2.53 (s, 3H, CH_3_), 7.60–8.64 (m, 14H, Ar-H), 8.84 (s, 1H, pyrazole-H5), 9.39 (s, 1H, CH-aliphatic, CH=N), 11.34, 11.64 (s, 2H, 2NH); ^13^C-NMR (DMSO-d_6_): δ 9.8, 117.7, 119.9, 123.5, 126.2, 127.7, 128.4, 129.3, 131.6, 133.5, 135.9, 139.7, 143.3, 143.6, 147.9, 170.2; MS *m*/*z* (%): 637 (M^+^, 25). Anal. Calcd for C_28_H_21_BrN_10_OS (637.56): C, 53.77; H, 3.38; N, 22.39. Found C, 53.82; H, 3.33; N, 22.32%.

2-(2-(3-(1-(4-bromophenyl)-5-methyl-1*H*-1,2,3-triazol-4-yl)-1-phenyl-1*H*-pyrazol-4-yl)methylene)hydrazinyl)-5-(2-(p-tolyl)hydrazono)thiazol-4(5H)-one (8b) 

Yellow solid; (82% yield); mp 175–177 °C (Ethanol); IR (KBr): *v*/cm^−1^ 3432, 3251 (2NH), 3101, 2930 (CH), 1697(C=O), 1610(C=C), 1600 (C=N); ^1^H-NMR (DMSO-d_6_): δ 2.46 (s, 3H, CH_3_), 2.67 (s, 3H, CH_3_), 7.37–8.56 (m, 13H, Ar-H), 8.81 (s, 1H, pyrazole-H5), 9.30 (s, 1H, CH-aliphatic, CH=N), 11.55 (s, broad, 2H, 2NH); ^13^C-NMR (DMSO-d_6_): δ 9.6, 117.7, 119.9, 123.5, 126.2, 127.7, 128.4, 129.3, 131.6, 133.5, 135.9, 139.7, 143.3, 143.6, 147.9, 170; MS *m/z* (%): 639 (M^+^, 57). Anal. Calcd for C_29_H_23_BrN_10_OS (639.54): C, 54.46; H, 3.63; N, 21.90 Found C, 54.52; H, 3.59; N, 21.82%. 2-(2-(3-(1-(4-bromophenyl)-5-methyl-1*H*-1,2,3-triazol-4-yl)-1-phenyl-1*H*-pyrazol-4-yl)methylene)hydrazinyl)-5-(2-(4-chlorophenyl)hydrazono)thiazol-4(5H)-one(8c)

Yellow solid; (73% yield); mp 181–183 °C (Ethanol); IR (KBr): *v*/cm^−1^ 3425, 3166 (2NH), 3110, 2974 (CH), 1697 (C=O), 1610(C=C), 1592 (C=N); ^1^H-NMR (DMSO-d_6_): δ 2.53 (s, 3H, CH3), 7.51–8.65 (m, 13H, Ar-H), 8.84 (s, 1H, pyrazole-H5), 9.31 (s, 1H, CH-aliphatic, CH=N), 11.59, 11.73 (s, 2H, 2NH); ^13^C-NMR (DMSO-d_6_): δ 9.6, 117.7, 119.9, 123.5, 126.2,127.7, 128.4, 129.3, 131.6, 133.5, 135.9, 139.7, 143.3, 143.6, 147.9, 170; MS *m*/*z* (%):659 (M^+^, 73). Anal. Calcd for C_28_H_20_BrClN_10_OS (659.95): C, 50.96; H, 3.05; N, 21.22. Found C, 50.91; H, 3.01; N, 21.17%.

2-(2-(3-(1-(4-bromophenyl)-5-methyl-1*H*-1,2,3-triazol-4-yl)-1-phenyl-1*H*-pyrazol-4-yl)methylene)hydrazinyl)-5-(2-(4-nitrophenyl)hydrazono)thiazol-4(5H)-one (8d)

Yellow solid; (68% yield); mp 152–154 °C (Ethanol); IR (KBr): *v*/cm^−1^ 3423, 3265 (2NH), 3125, 2970 (CH), 1705 (C=O), 1620(C=C), 1600 (C=N); ^1^H-NMR (DMSO-d_6_): δ 2.50 (s,3H, CH_3_), 7.45-8.57 (m, 13H, Ar-H), 8.77 (s, 1H, pyrazole-H5), 9.29 (s, 1H, CH-aliphatic, CH=N), 11.56 (s, broad, 2H, 2NH); ^13^C-NMR (DMSO-d6): δ 9.8, 117.9, 119.9, 123.7, 126.4, 127.7, 128.4, 129.3, 131.6, 133.5, 135.9,139.7, 143.5, 143.8, 147.9, 170.4; MS *m*/*z* (%): 670 (M^+^, 100). Anal. Calcd for C_28_H_20_BrN_11_O_3_S (670.51): C, 50.16; H, 3.01; N, 22.98 Found C, 50.12; H, 2.97; N, 22.92%.

*Alternate method for **8a***–***d***: Synthesis of 2-(2-((3-(1-(4-bromophenyl)-5-methyl-1*H*-1,2,3-triazol-4-yl)-1-phenyl-1*H*-pyrazol-4-yl)methylene)hydrazinyl)thiazol-4(5H)-one (9) To a mixture of thiosemicarbazone **3** (4.8 g, 10mmol) and anhydrous sodium acetate (0.33 g, 4 mmol) in EtOH (20 mL), ethyl chloroacetate (1.22 g, 10 mmol) was added, and then mixture was refluxed for 6–8 h (monitored by TLC), and then left to cool. The solid product was filtered off, washed with water and recrystallized from AcOH to afford the thiazolone derivative **9** as brown solid (75% yield); mp 222–224 °C (AcOH); IR (KBr): *v*/cm^−1^ 3423 (NH), 3129, 2917 (CH), 1712(C=O), 1620(C=C), 1600 (C=N); ^1^H-NMR (DMSO-d_6_): δ 2.50 (s, 3H, CH_3_), 4.2(s, 2H, CH_2_), 7.45–8.57 (m, 9H, Ar-H), 8.72 (s, 1H, pyrazole-H5), 9.29(s, 1H, CH-aliphatic, CH=N), 11.56 (s, 1H, NH); ^13^C-NMR (DMSO-d_6_): δ 9.6, 36.4, 119.6, 123.5, 127.2, 128.4, 129.3, 131.6, 133.5, 135.9, 139.7, 143.3, 143.6, 158.3, 176.4; MS *m*/*z* (%): 521 (M^+^, 100). Anal. Calcd for C_22_H_17_BrN_8_OS (521.40): C, 50.68; H, 3.29; N, 21.49 Found C, 50.73; H, 3.26; N, 21.45%.

*Coupling of thiazole 9 with arenediazonium chlorides:* A solution of **9** (0.10 g, 2 mmol) in ethanol (20 mL), cooled to 0–5 °C in an ice bath, was added portion-wise to a cold solution of arenediazonium chloride [prepared by diazotizing aniline derivatives (2 mmol) dissolved in hydrochloric acid (6 M, 2 mL) with a solution of sodium nitrite (0.14 g, 2 mmol) in water (3 mL)]. After the complete addition of the diazonium salt, the reaction mixture was stirred for a further 30 min in an ice bath. The solid that separated was filtered off, washed with water and finally recrystallized from EtOH to give products proved to be identical in all respects (mp, mixed mp and IR spectra) with compounds **8a**–**d**, obtained from reaction of **3** with **7a**–**d**.

2-(2-((3-(1-(4-bromophenyl)-4-methyl-1*H*-1,2,3-triazol-5-yl)-1-phenyl-1*H*-pyrazol-4-yl)methylene)hydrazinyl)-4-phenylthiazole (13a)

Brown solid; (68% yield); mp 210–112 °C (Acetic acid); IR (KBr): *v*/cm^−1^ 3429 (NH), 3025, 2922 (CH), 1600 (C=N); ^1^H-NMR (DMSO-d_6_): δ 2.49 (s,3H, CH_3_), 7.26–8.48 (m, 14H, Ar-H), 8.67(s,1H, thazole-H5), 8.81(s, 1H, pyrazole-H5), 8.94(s, 1H, CH-aliphatic, CH=N), 11.52 (s, 1H, NH); δ 9.6, 119.6, 123.5, 126.2, 128.4, 129.3, 131.6, 133.5, 139.7, 143.3, 143.6, 147.9, 165.3; MS *m*/*z* (%):581 (M^+^, 73). Anal. Calcd for C_28_H_21_BrN_8_S (581.50): C, 57.83; H, 3.64; N, 19.27 Found C, 57.92; H, 3.59; N, 19.22%.

2-(2-((3-(1-(4-bromophenyl)-4-methyl-*1H*-1,2,3-triazol-5-yl)-1-phenyl-*1H*-pyrazol-4-yl)methylene)hydrazinyl)-4-(4-chlorophenyl)thiazole (13b)

Brown solid; (68% yield); mp 228–230 °C (Acetic acid); IR (KBr): *v*/cm^−1^ 3435 (NH), 3029, 2927 (CH), 1610 (C=N); ^1^H-NMR (DMSO-d_6_): δ 2.59(s,3H, CH_3_), 7.23–8.49 (m, 13H, Ar-H), 8.76(s,1H, thazole-H5), 8.87(s, 1H, pyrazole-H5), 8.98 (s, 1H, CH-aliphatic, CH=N), 11.71 (s, 1H, NH); δ 9.8, 120.0, 123.7, 126.2, 128.4, 129.3, 131.6, 133.5, 139.7, 143.3, 143.6, 147.9, 165.5; MS *m*/*z* (%): 615 (M^+^, 30). Anal. Calcd for C_28_H_20_BrClN_8_S (615.94): C, 54.60; H, 3.27; N, 18.19 Found C, 54.69; H, 3.25; N, 18.12%.

2-(2-((3-(1-(4-bromophenyl)-5-methyl-*1H*-1,2,3-triazol-4-yl)-1-phenyl-*1H*-pyrazol-4-yl)methylene)hydrazinyl)-4-(pyridin-2-yl)thiazole (14)

Yellow solid; (62% yield); mp 192–194 °C (Acetic acid); IR (KBr): *v*/cm^−1^ 3395 (NH), 3029, 2921 (CH), 1600 (C=N); ^1^H-NMR (DMSO-d_6_): δ 2.49 (s,3H, CH_3_), 7.37–8.59 (m, 8H, Ar-H), 8.72 (s,1H, thazole-H5), 8.91(s, 1H, pyrazole-H5), 9.01(s, 1H, CH-aliphatic, CH=N), 11.52 (s, 1H, NH); ^13^C-NMR (DMSO-d_6_): δ 9.6, 119.6, 123.5, 126.2, 128.4, 129.3, 131.6, 133.5, 135.9, 139.7, 143.3, 143.6, 147.9, 165.3; MS *m*/*z* (%): 582 (M^+^, 72). Anal. Calcd for C27H20BrN9S (582.48): C, 55.67; H, 3.46; N, 21.64 Found C, 55.74; H, 3.42; N, 21.61%.

2-(2-(2-((3-(1-(4-bromophenyl)-5-methyl-1*H*-1,2,3-triazol-4-yl)-1-phenyl-*1H*-pyrazol-4-yl)methylene)hydrazinyl)thiazol-4-yl)-*3H*-benzochromen-3-one (15)

Yellow solid; (69% yield); mp 122–224 °C (EtOH); IR (KBr): *v*/cm^−1^ 3384 (NH), 3027, 2915 (CH), 1605 (C=N); ^1^H-NMR (DMSO-d_6_): δ 2.50 (s,3H, CH_3_), 7.41–8.45 (m, 15H, Ar-H), 8.49 (s, 1H, coumarine-H4), 8.81 (s,1H, pyrazole-H5), 8.93 (s, 1H, thiazole-H5), 9.40 (s, 1H, CH-aliphatic, CH=N), 11.53 (s, 1H, NH); ^13^C-NMR (DMSO-d_6_): δ 9.6, 114.3, 115.6, 123.5, 126.2, 128.4, 129.3, 131.5, 133.5, 135.9, 139.7, 143.3, 143.6, 147.9, 151.2, 162.9, 172.2; MS *m*/*z* (%): 699 (M^+^, 39). Anal. Calcd for C_35_H_23_BrN_8_O_2_S (699.59): C, 60.09; H, 3.31; N, 16.02 Found C, 60.17; H, 3.27; N, 15.96%.

### 4.2. Computational Studies

In this work, the binding of newly synthesized compounds to Rho6 was theoretically investigated using a computer-based docking approach. The X-ray crystal structure of the target Rho6 is retrieved from the RCSB Protein Data Bank web server (www.rcsb.org/pdb/) [35]. The two-dimensional chemical structures of the compounds are drawn using Chem Draw Ultra 0.7, and then converted to SDF format using Open Babel 2.4.1 tool [36]. The docking area is selected by generating a grid box centered at x, y and z coordinates. The in silico docking study between the newly compounds and the binding site pocket of the target is carried out using a PyRx 8.0 tool [37]. In a docking simulation, the compounds are assumed to be flexible, and the docking tool is allowed to rotate all the rotatable bonds of the compounds to obtain the best and optimized conformer of the docked molecule. The Lamarckian genetic algorithm (LGA) is used as a scoring function to calculate the different conformers of each docked compound [38]. Prediction of pharmacokinetics and physicochemical parameters of the target compounds plays an integral role in drug discovery [9]. The evaluation of drug-likeness properties for all compounds is performed using the SwissADME and Mol inspiration web-based servers [39,40]. These tools are used to evaluate the compounds based on Lipinski’s rule of five (RO5), which states that an active oral drug should qualify the following parameters: the molecular mass MW should be ≤500 g/mol; the logarithm of partition coefficient between *n*-octanol and water log P should be <5; the number of hydrogen bond acceptors should be nOH 2.0–20.0; the number of hydrogen bond donors nOHNH should be 0.0–6.0; and the number of rotatable bonds should be ≤10 [41]. Compounds violating more than one of these rules may have bioavailability problems.

### 4.3. Biological Activity

The cytotoxic evaluation of the synthesized compounds was carried out at the Regional Center for Mycology and Biotechnology at Al-Azhar University, Cairo, Egypt, according to the reported methods [42,43].

## Figures and Tables

**Table 1:** Molecular docking results for the screened compounds and Rho6 protein.

	2D Structure	BE kcal.mol^−1^	Docked Complex (Amino Acid-Ligand) Interactions	Bond Length (Å)
**1**	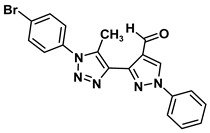	−6.8	**Arene-cation interaction** Lys106:NZ-compound 1	4.95
			**Arene-sigma interaction** Lys106:CG-compound 1	3.46
**3**	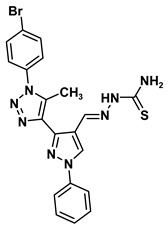	−7.2	**Arene-cation interaction** Arg96:NH1-compound 3	3.98
**5a**	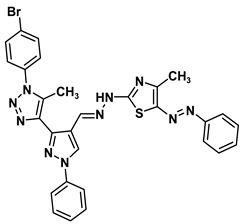	−8.2	**Arene-cation interaction** Arg96:NH1-compound 5a	3.95
**5b**	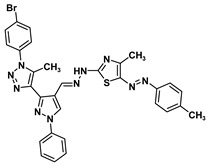	−9.4	**H-bond interaction** Gln158:O-compound 5b	1.97
			**Arene-cation interaction** Arg108:NH1---compound 5b	4.02
**5c**	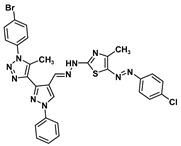	−9.0	**H-bond interaction** Gln158:N-compound 5c	2.35
			**H-bond interaction** Leu159:N-compound 5c	2.20
**5d**	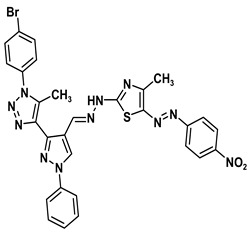	−9.1	**H-bond interaction** Gln158:N-compound 5d Leu159:N-compound 5d Gly160:N-compound 5d Ala161:O-compound 5d	2.43 2.59 1.93 1.99
			**Arene-cation interaction** Lys157:NZ-compound 5d	5.49
**8a**	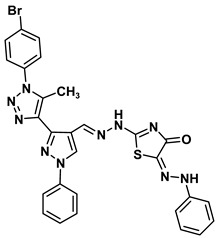	−6.5	**H-bond interaction** Ser64:OG-compound 8a	2.10
			**H-bond interaction** Trp66:NE1-compound 8a	1.96
**8b**	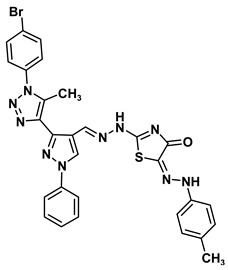	−9.9	**H-bond interaction** Gln158:N-compound 8b Leu159:N-compound 8b Gln158:O-compound 8b	3.10 2.98 1.94
**8c**	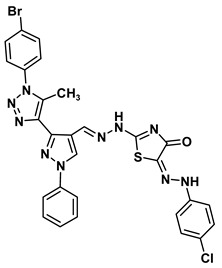	−8.9	**H-bond interaction** Arg108:NE-compound 8c Arg108:NH1-compound 8c Val120:N-compound 8c Leu121:N-compound 8c	2.61 2.99 1.88 2.63
			**Arene-sigma interaction** Val120:CG1-compound 8c	3.63
**8d**	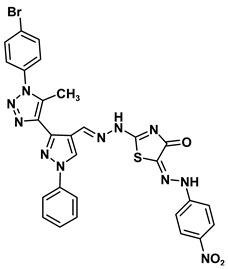	−9.8	**H-bond interaction** Gly160:N-compound 8d Gly160:N-compound 8d Gln158:O-compound 8d	2.98 2.81 1.30
**13a**	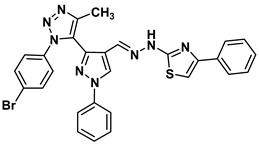	−7.7	**H-bond interaction** Ser95:OG-compound 13a Glu138:OE1-compound 13a	2.30 2.15
			**Arene-cation interaction** Arg96:NH1---compound13a	4.10
**13b**	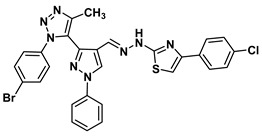	−7.8	**H-bond interaction** Asp132:OD2-compound 13b	2.99
**14**	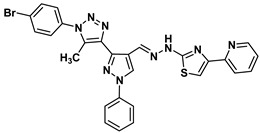	−7.9	**H-bond interaction** Ser95:OG-compound 14	2.95
			**Arene-cation interaction** Arg96:NH1-compound14	3.88
**15**	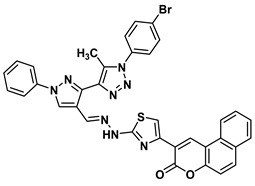	−9.2	**Arene-cation interaction** Lys15:NZ-compound 15 Lys15:NZ-compound 15	5.74 5.50

B.E, estimated free binding energy.

**Table 2:** List of ADME and physicochemical properties of the title compounds **1**–**15**.

	MW (g/mol)	BBB^+^	Caco2^+^	HIA^+^	logp	TPSA A^2^	nON	nOHNH	RBs	N Violations	AMES Toxicity	Carcinogenicity
	180–500	−3 to 1.2	< 25 poor > 500 great	< 25 poor >80 high	<5	≤ 140	2.0–20.0	0.0–6.0	≤ 10	< 5	Nontoxic	Non carcinogenic
**1**	408.26	0.98	67.17	98.94	3.15	65.61	6	0	4	0	Nontoxic	Noncarcinogenic
**3**	481.38	0.97	65.20	96.57	3.31	98.96	8	3	6	0	Nontoxic	Noncarcinogenic
**5a**	623.54	0.98	82.20	92.98	6.24	110.56	10	1	8	2	Nontoxic	Noncarcinogenic
**5b**	637.57	0.98	82.51	92.96	6.69	110.56	10	1	8	2	Nontoxic	Noncarcinogenic
**5c**	657.99	0.97	82.77	92.93	6.92	110.56	10	1	8	2	Nontoxic	Noncarcinogenic
**5d**	668.54	0.97	82.69	92.95	6.20	156.38	13	1	9	3	Nontoxic	Noncarcinogenic
**8a**	625.52	0.98	83.26	97.32	4.74	127.29	11	2	8	1	Nontoxic	Noncarcinogenic
**8b**	639.54	0.98	83.64	97.35	5.19	127.29	11	2	8	2	Nontoxic	Noncarcinogenic
**8c**	659.96	0.98	83.67	97.96	5.42	127.29	11	2	8	2	Nontoxic	Noncarcinogenic
**8d**	670.51	0.98	83.93	97.98	4.70	173.11	14	2	9	2	Nontoxic	Noncarcinogenic
**13a**	581.50	0.97	79.71	92.98	5.38	85.83	8	1	7	2	Nontoxic	Noncarcinogenic
**13b**	615.95	0.97	82.80	94.06	6.06	85.83	8	1	7	2	Nontoxic	Noncarcinogenic
**14**	582.49	0.98	78.83	92.98	4.23	98.72	9	1	7	1	Nontoxic	Noncarcinogenic
**15**	699.59	0.98	85.73	88.62	6.54	116.04	10	1	7	2	Nontoxic	Noncarcinogenic

MW: Molecular Weight; BBB^+^: Blood-Brain Barrier; Caco^2+^, Caco-2: Permeability; HIA^+^: %Human Intestinal Absorption; logp: logarithm of partition coefficient between *n*-octanol and water; TPSA^2^: topological polar surface area; nON: number of hydrogen bond acceptors; nOHNH: number of hydrogen bond donors; RBs: number of rotatable bond.

**Table 3:** Antiproliferative activity of the new derivatives towards liver (HepG2) and normal (BALAB/3T3) cell lines.

Comp. N^o^	R	HepG2 IC_50_ ± SD [µg/mL]	BALAB/3T3 IC50 ± SD [µg/mL]	General Structure
**Doxorubicin**		3.56 ± 0.84	1.86 ± 0.07	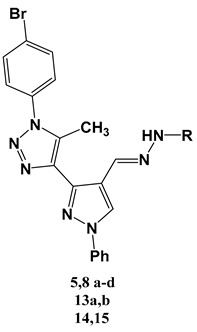
**5a**	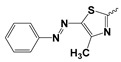	35.64 ± 6.07	Nd	
**5b**	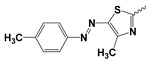	2.30 ± 2.72	Nd	
**5c**	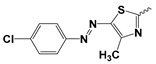	32.32 ± 6.09	10.09 ± 0.23	
**5d**	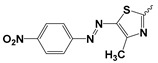	36.79 ± 15.70	Nd	
**8a**	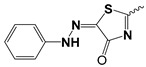	49.05 ± 5.37	16.72 ± 3.24	
**8b**	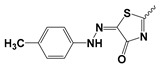	11.83 ± 0.29	Nd	
**8c**	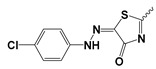	43.30 ± 14.77	Nd	
**8d**	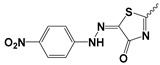	18.24 ± 0.08	Nd	
**13a**	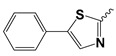	27.34 ± 6.14	Nd	
**13b**	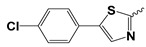	30.26 ± 3.05	43.23 ± 2.36	
**14**	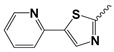	19.75 ± 9.37	Nd	
**15**	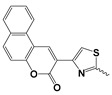	29.61 ± 2.74	Nd	

## Data Availability

All data generated or analyzed during this study are included in this manuscript.

## Supplementary Materials

Figure S1: 2D and 3D representations of Rho6-compound complexes.

## Sample Availability

Samples of the compounds are not available from the authors.
