# Supplementary material for: Synthesis, Molecular Docking Studies and In Silico ADMET Screening of New Heterocycles Linked Thiazole Conjugates as Potent Anti-Hepatic Cancer Agents
Source: Molecules. 2021 Mar 18;26(6):1705. doi: 10.3390/molecules26061705 (PMC8003218; doi:10.3390/molecules26061705)
Supplement: Supplementary file 1 [file molecules-26-01705-s001.zip › molecules-1128022-supplementary.pdf]

Compound **5c.**

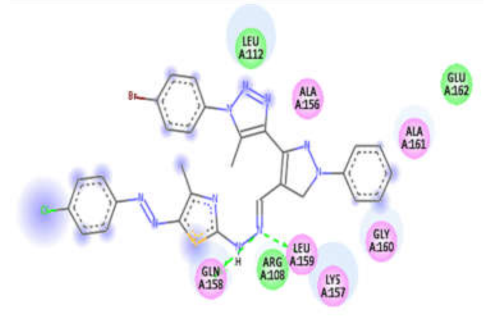

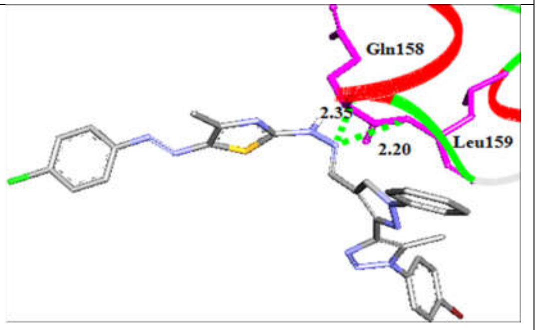

Compound **5d**

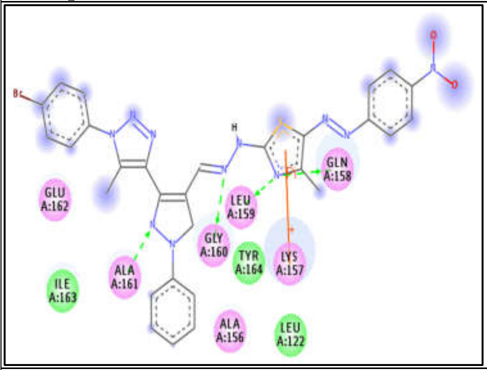

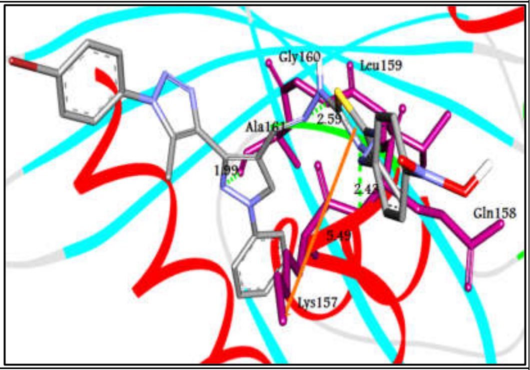

Compound **8a**

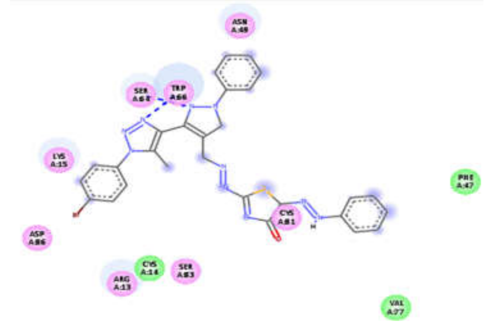

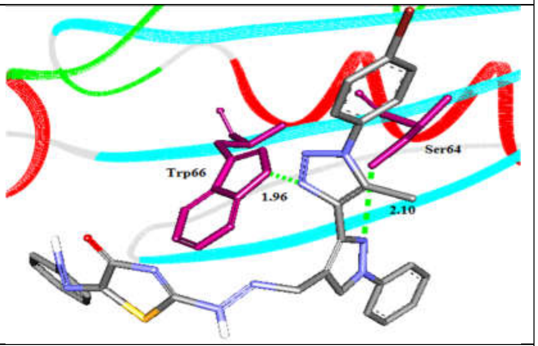

Compound **8b**

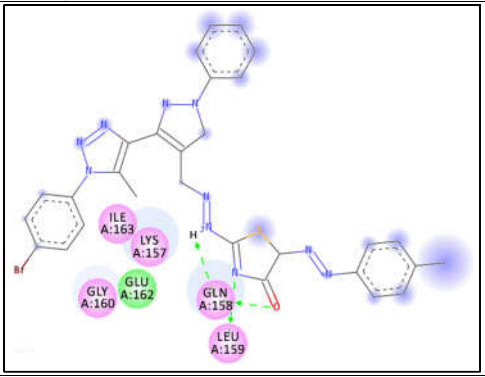

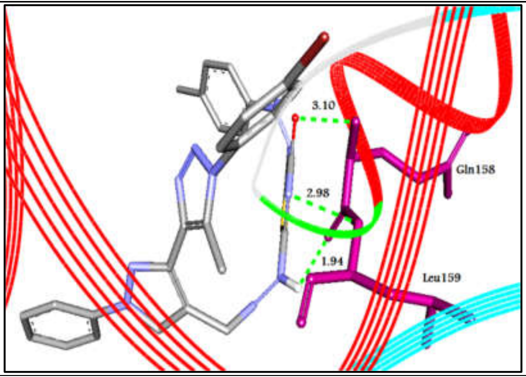

Compound **8c**

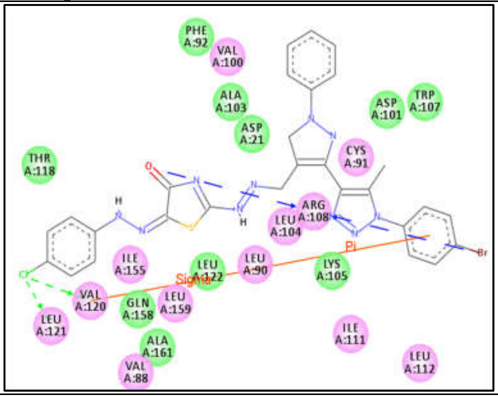

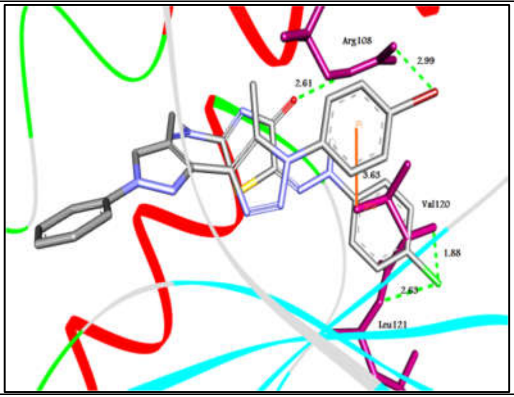

Compound **8d**

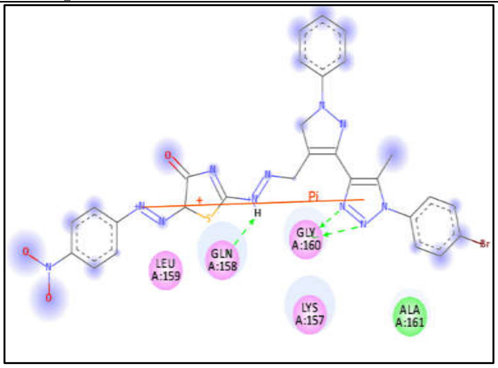

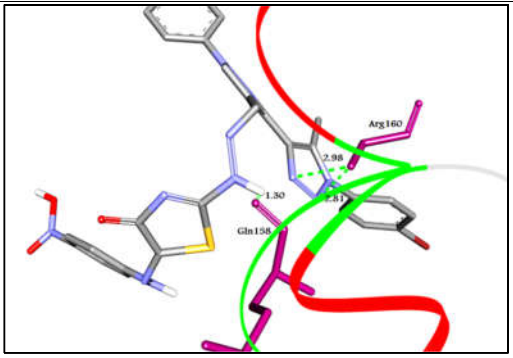

Compound **13a**

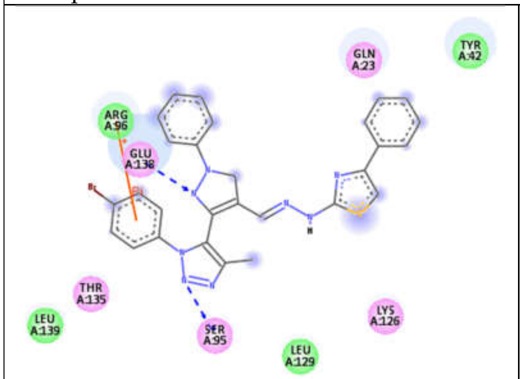

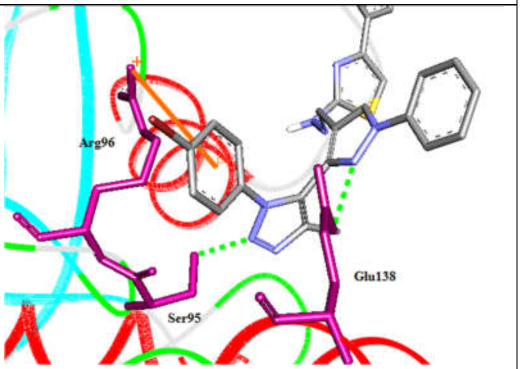

Compound **13b**

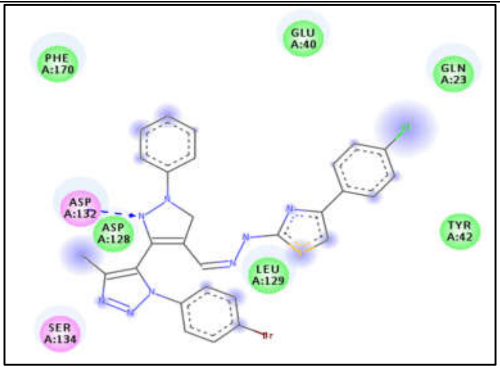

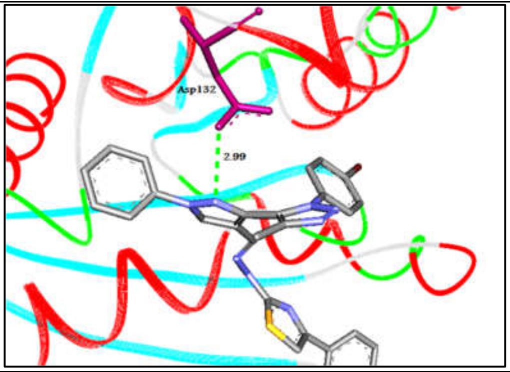

Compound **14**

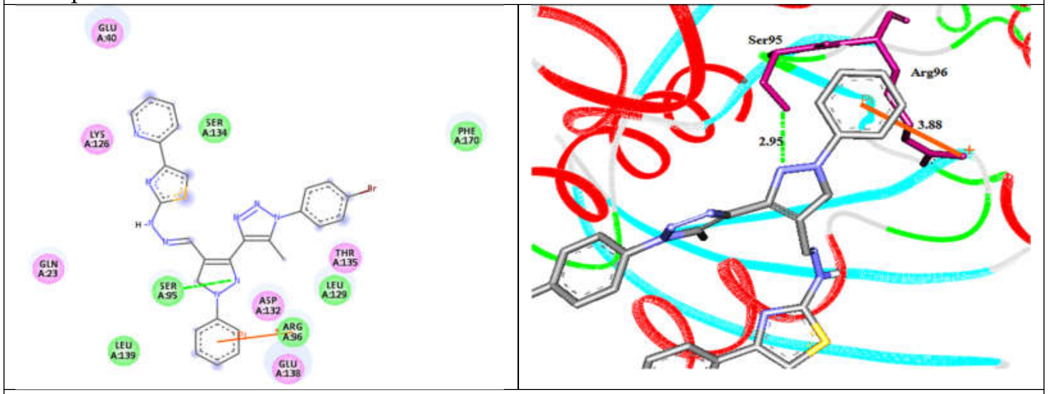

Compound **15**

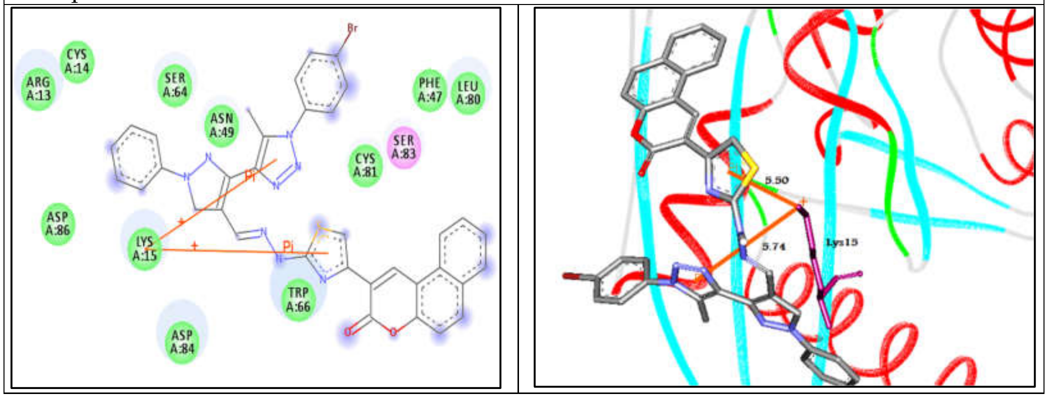

Figure 1. 2D and 3D representations of Rho6-compound complexes. Hydrogen bonds are represented in green and blue dotted lines, while π-stacking are shown in orange lines.
